# Sigma-1 Receptor Agonists Acting on Aquaporin-Mediated H_2_O_2_ Permeability: New Tools for Counteracting Oxidative Stress

**DOI:** 10.3390/ijms22189790

**Published:** 2021-09-10

**Authors:** Giorgia Pellavio, Giacomo Rossino, Giulia Gastaldi, Daniela Rossi, Pasquale Linciano, Simona Collina, Umberto Laforenza

**Affiliations:** 1 Department of Molecular Medicine, Human Physiology Unit, University of Pavia, I-27100 Pavia, Italy; giorgia.pellavio@gmail.com (G.P.); giulia.gastaldi@unipv.it (G.G.); 2 Department of Drug Sciences, Medicinal Chemistry and Pharmaceutical Technology Section, University of Pavia, I-27100 Pavia, Italy; giacomo.rossino01@universitadipavia.it (G.R.); daniela.rossi@unipv.it (D.R.); pasquale.linciano@unipv.it (P.L.); simona.collina@unipv.it (S.C.); 3 Centre for Health Technology (CHT), University of Pavia, I-27100 Pavia, Italy

**Keywords:** peroxiporins, oxidative stress, hydrogen peroxide, water channels, Sigma1 receptors, Sigma1 receptor modulators, neurodegenerative diseases

## Abstract

Sigma1 Receptor (S1R) is involved in oxidative stress, since its activation is triggered by oxidative or endoplasmic reticulum stress. Since specific aquaporins (AQP), called peroxiporins, play a relevant role in controlling H_2_O_2_ permeability and ensure reactive oxygen species wasted during oxidative stress, we studied the effect of S1R modulators on AQP-dependent water and hydrogen peroxide permeability in the presence and in the absence of oxidative stress. Applying stopped-flow light scattering and fluorescent probe methods, water and hydrogen peroxide permeability in HeLa cells have been studied. Results evidenced that S1R agonists can restore water permeability in heat-stressed cells and the co-administration with a S1R antagonist totally counteracted the ability to restore the water permeability. Moreover, compounds were able to counteract the oxidative stress of HeLa cells specifically knocked down for S1R. Taken together these results support the hypothesis that the antioxidant mechanism is mediated by both S1R and AQP-mediated H_2_O_2_ permeability. The finding that small molecules can act on both S1R and AQP-mediated H_2_O_2_ permeability opens a new direction toward the identification of innovative drugs able to regulate cell survival during oxidative stress in pathologic conditions, such as cancer and degenerative diseases.

## 1. Introduction

Hydrogen peroxide (H_2_O_2_) is the most abundant and stable reactive oxygen species (ROS) in living cells [1]. At low physiological concentrations, H_2_O_2_ may act as signaling molecule and is involved in various physiological processes through autocrine or paracrine mechanisms [1]. Conversely, when H_2_O_2_ accumulates within cells, due to a disbalance between ROS production and scavenging, it is responsible for oxidative stress [2,3,4,5,6]. When oxidative stress occurs, the cells dispose of H_2_O_2_ either by intracellular antioxidant systems or by outflow through the plasma membrane. H_2_O_2_ crosses the biological membranes through a diffusion-facilitating channel mechanism mediated by Aquaporins (AQPs) [1,3,7]. AQPs are integral membrane proteins forming channels in the biological membranes, and are mainly involved in the transport of water and small molecules (i.e., ammonia, glycerol) [8,9]. To date, five AQP isoforms (AQP3 [10,11,12,13], AQP5 [14,15], AQP8 [4,16], AQP9 [17] and AQP11 [18]) showed additional H_2_O_2_ permeability and they are therefore called peroxiporins. The control of peroxiporins-mediated H_2_O_2_ permeability seems to have a great importance in regulating cell signaling and survival during oxidative stress [4,19,20,21]. For living cells, the functioning of peroxiporins is critical to ensure ROS wasting and is considered an antioxidant system. Various cellular stress conditions, including heat and incubation with H_2_O_2_, reduce the AQP-mediated H_2_O_2_ transport [4,19,20,21]. Recently, a number of natural antioxidants, such as flavonoids, flavanones and terpenoids, has been identified as AQPs modulators [20]. The addition of such compounds, during or after heat-treatment, is able to prevent or reverse the AQPs permeability [20]. This suggests the possibility to chemically modulate the pore gating of peroxiporins, supporting the idea that AQPs are druggable targets.

These findings open a new direction to the development of novel therapeutic treatments to regulate cell signaling and survival during oxidative stress in normal and pathologic conditions, such as cancer and degenerative diseases [22,23]. 

Another physiological cellular response to oxidative stress requires the activation of the sigma-1 receptor (S1R). Despite intensive research since its identification in 1976, it still represents an enigmatic target, whose molecular functions are not fully understood yet. Nowadays, the most widely accepted model describes it as a ligand-operated chaperone able to interact with a plethora of partner proteins. 

S1R is a chaperone protein, mainly expressed in mitochondria-associated endoplasmic reticulum (ER) membranes [24]. Interestingly, it has been demonstrated that also AQP8 and AQP11 are localized in mitochondria [25,26] and ER [18], respectively and possess H_2_O_2_ permeability.

Upon activation, S1R modulates diverse signaling pathways connected to cell survival and excitability, including calcium homeostasis, reduction of glutamate release, ROS, nitric oxide (NO), microglial activity, and upregulation of antiapoptotic genes (i.e., Bcl-2) [27]. This suggests that modulation of S1R is an effective strategy to counteract oxidative stress by reducing levels of reactive oxygen species, although mechanisms responsible for the antioxidant effects exerted by S1R have not been completely clarified yet [28,29,30]. Moreover, S1R provides additional layers of protection during ER stress thanks to its chaperoning activities against a plethora of diverse client proteins [24,31]. For this reason, S1R has been recognized as a promising therapeutic target and is currently being investigated for several complex multifactorial pathologies, such as neurodegeneration and neuropathic pain [32,33,34]. As a result, S1R modulators are under investigation for treating several diseases involving oxidative stress [35] such as cardiovascular diseases, neurodegenerative disorders, diabetes, ischemia/reperfusion, Alzheimer’s [36,37,38] and CNS inflammatory conditions associated with cocaine and HIV [38,39]. 

As a part of our current medicinal chemistry research on S1R [40,41,42,43,44], we have recently discovered a series of ligands endowed with nanomolar S1R binding affinity and with antioxidant activity in cellular models [20,43,45,46,47]. Of note, the potent S1R agonist identified in our lab, called RC33, is currently under investigation in vivo for its potential against Amyotrophic Lateral Sclerosis and for recovery of the damage of Spinal Cord Injury [40].

**Figure 1:**
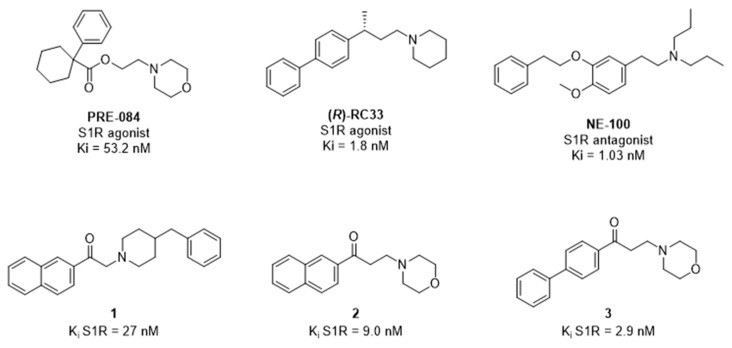
Chemical structures of S1R ligands tested in this study to evaluate their AQPs-mediated antioxidant effect.

Starting from the evidence that AQP8 and AQP11 are localized in mitochondria [25,26] and endoplasmic reticulum (ER) [18], respectively, and that S1R is also mainly expressed in mitochondria-associated ER membranes [24], in the present paper, we studied the potential of S1R modulators as AQPs interfering compounds. Briefly, we studied the in vitro effect of the well-established S1R agonists PRE084 and RC33, and of the S1R antagonist NE100, on AQPs. The investigation was then extended to three in-house developed S1R ligands (compounds **1**–**3**, Figure 1), for which additional intrinsic antioxidant properties have already been demonstrated [45]. Lastly, to study if the antioxidant effect of S1R modulators is mediated by AQPs, experiments with S1R-knockdown (KD) cells have been performed.

Considering that the osmotic water permeability of AQPs is indicative of H_2_O_2_ permeability [4,19,48], that HeLa cells express different AQP proteins (AQP1, AQP3, AQP8 and AQP11) and that they have been already used to test the antioxidant capacity of some natural compounds [20], in this paper the effects of S1R agonists were evaluated in HeLa cells under eustress and oxidative stress conditions. 

## 2. Results

For evaluating water and hydrogen peroxide permeability in HeLa cells, in this study we used a cheap, very sensitive and reproducible method based on stopped-flow light scattering. Moreover, heat was used as a physiological stressor able to reduce water permeability in cells, as reported by previous works [4,20].

**Figure 2:**
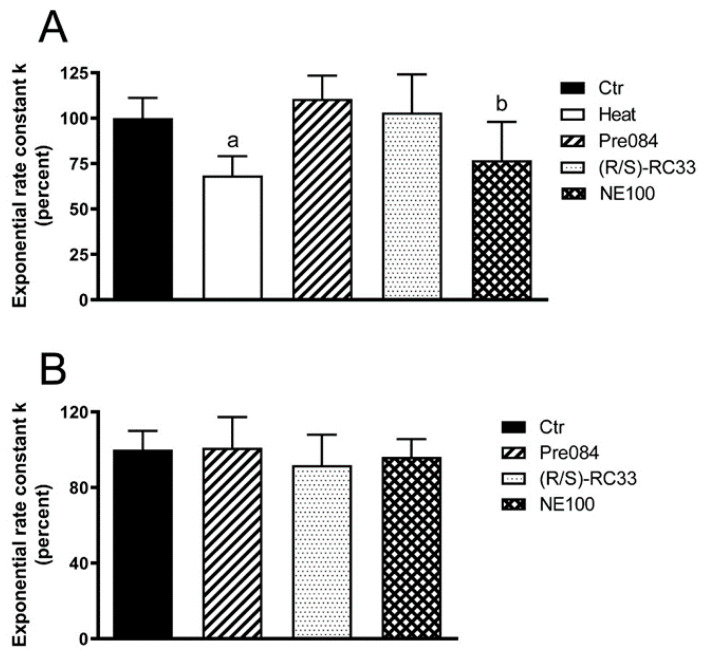
Effect of S1R modulators on the water permeability of HeLa cells in heat-stress conditions (**A**) and in normal non-stressed conditions (**B**). Bars represent the osmotic water permeability of HeLa cells expressed as percent of the exponential rate constant k. Values are means ± SD of 4–15 single shots (time course curves) for each of 4–6 different experiments. (**A**) HeLa cells were exposed to a 150 mOsm osmotic gradient in different conditions: untreated cells (Controls, Ctr); cells treated at 42 °C for 3 h (heat-stressed, Heat); heat-stressed cells pre-treated with test compounds (Pre084, (*R*/*S*)-RC33 and NE100) at 20 μM final concentration. (**B**) HeLa cells were incubated at 21 °C for 3 h with the compounds at 20 μM final concentration and, successively, were exposed to a 150 mOsm osmotic gradient. Controls (Ctr) indicate cells incubated without compounds. a, *p* < 0.05 vs. Ctr, Pre084, (*R*/*S*)-RC33; b, *p* < 0.05 vs. Ctr (Repeated measures ANOVA, followed by Newman-Keuls’s *Q* test).

The well characterized S1R agonists PRE084 and RC33 ((*R*/*S*), racemic and (*R*)-configured) and the S1R antagonist NE100 have been tested in a first set of experiments at the fixed concentration of 20 µM. Results reported in Figure 2 clearly evidenced a different behavior of S1R modulators, depending on their agonist/antagonist profile. 

**Figure 3:**
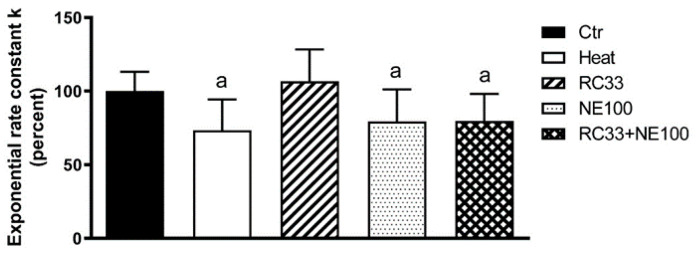
Effect of the NE100 and (*R/S*)-RC33 co-administration on the water permeability of HeLa cells in heat-stress condition. Comparison between untreated cells (Controls, Ctr), cells treated at 42 °C for 3 h (heat-stressed, Heat), heat-stressed cells pre-treated separately with NE100 and (*R/S*)-RC33 at 20 μM and heat-stressed cells pre-treated simultaneously with NE100 and (*R/S*)-RC33. Bars represent the osmotic water permeability of HeLa cells expressed as percent of the exponential rate constant k. Values are means ± SD of 4–15 single shots (time course curves) for each of 4–6 different experiments. a, *p* < 0.05 vs. Ctr, (*R/S*)-RC33 (Repeated measures ANOVA, followed by Newman-Keuls’s *Q* test).

Although the treatment of cells under normal conditions with the S1R modulators had no effect on water permeability (Figure 2B), in heat-stressed cells the pre-treatment with the S1R agonists PRE084, (*R*/*S*)-RC33 and (*R*)-RC33, i.e., the most metabolically stable enantiomer [49] (see below), were able to restore water permeability. Conversely, the Sigma-1 antagonist NE100 did not produce any effect (Figure 2A). To assess if the modulation of water and hydrogen peroxide permeability in HeLa cells is mediated by S1R, a co-administration of NE100 with RC33, used as model agonist, was performed. As a result, the effect of RC33 was totally counteracted (Figure 3), thus confirming that the effect is S1R mediated.

**Figure 4:**
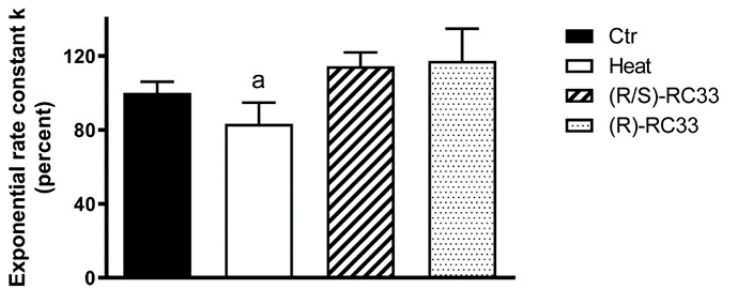
Effect of the RC33 stereochemistry on the water permeability of HeLa cells in heat-stress condition. Comparison between untreated cells (Controls, Ctr), cells treated at 42 °C for 3 h (heat-stressed, Heat), heat-stressed cells pre-treated with racemic (*R*/*S*)-RC33 and enantiopure (*R*)-RC33 at 20 μM final concentration. Bars represent the osmotic water permeability of HeLa cells expressed as percent of the exponential rate constant k. Values are means ± SD of 4–15 single shots (time course curves) for each of 4–6 different experiments. a, *p* < 0.05 vs. Ctr, (*R*/*S*)-RC33, (*R*)-RC33 (Repeated measures ANOVA, followed by Newman-Keuls’s *Q* test).

As a further step, we studied whether RC33 stereochemistry plays a role in the modulation of AQPs. Accordingly, we compared the effect of racemic RC33 with that of (*R*)-RC33, which was previously identified as the most metabolically stable enantiomer in our studies [49]. Racemic and (*R*)-configured RC33 showed a similar behavior in the experiments on water permeability (Figure 4). 

**Figure 5:**
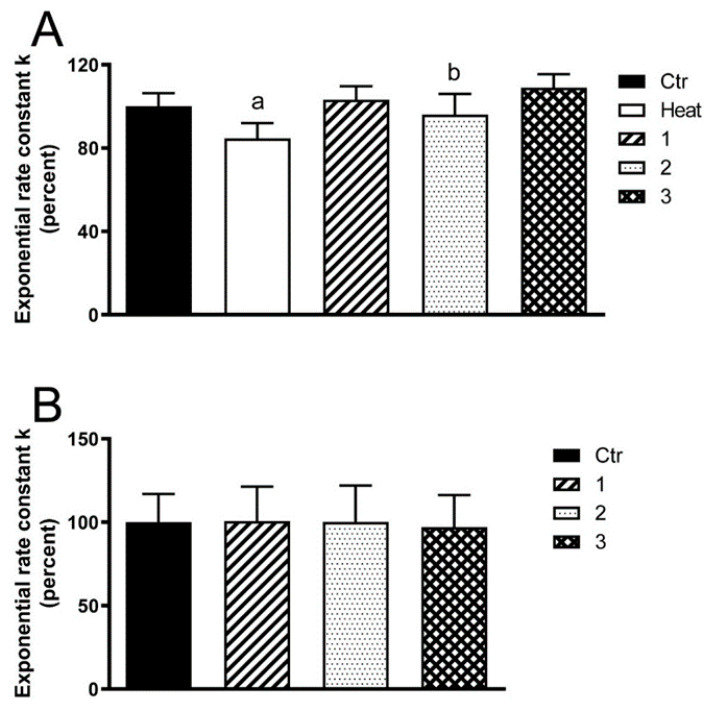
Effect of compounds **1**–**3** on the water permeability of HeLa cells in heat-stress condition (**A**) and in normal non-stressed condition (**B**). (**A**) Comparison between untreated cells (Controls, Ctr), cells treated at 42 °C for 3 h (heat-stressed, Heat), heat-stressed cells pre-treated with test compounds at 20 μM final concentration. (**B**) Effect of the compounds on water permeability of HeLa cells incubated at 21 °C for 3 h. Controls (Ctr) indicate cells incubated without compounds. Bars represent the osmotic water permeability of HeLa cells expressed as percent of the exponential rate constant k. Values are means ± SD of 4–15 single shots (time course curves) for each of 4–6 different experiments. a, *p* < 0.01 vs. Ctr, **1**, **2**, **3**; b, *p* < 0.05 vs. **3** (Repeated measures ANOVA, followed by Newman-Keuls’s *Q* test).

Once the profile of well-known S1R modulators was assessed in water permeability assays, we extended the investigation to compounds **1**–**3**, belonging to a library of aryl aminoalkyl ketones with S1R binding affinity and antioxidant properties [45]. Heat-stressed cells treated with compounds **1**–**3** displayed a restored water permeability (Figure 5A). HeLa cells treated with compound **3** had a significantly higher water permeability than those incubated with compound **2** (Figure 5A). The behavior is superimposable with that of well-established S1R agonists PRE084 and RC33 and once again, the treatment of the cells with our test compounds under normal conditions did not affect water permeability (Figure 5B). 

**Figure 6:**
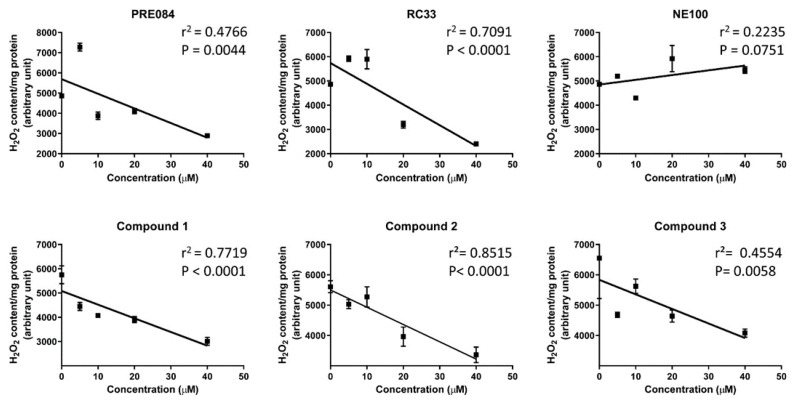
Dose–response effect of compounds Pre084, RC33, NE100, **1**, **2** and **3** on HeLa cells H_2_O_2_ content. HeLa cells were heat-stressed at 42 °C for 3 h and pre-treated with increasing concentrations of test compounds. H_2_O_2_ was measured using the CM-H2DCFDA reagent (see Materials and methods). Values represent mean ± SD (*n* = 4) and are expressed as H_2_O_2_ content (arbitrary unit) per mg protein. When not shown, SD were within the symbol area. Overall, linear regression (black line) is presented. *p* and r^2^ values are shown.

To exclude that the effect on AQPs is non-specific, the ability of tested compounds to reduce H_2_O_2_ content within the cell (Figure 6) at different concentrations have been evaluated. Results confirmed that only S1R agonists are effective in reduction of H_2_O_2_ levels and that all S1R agonists showed a dose-response effect. Nevertheless, a similar response per unit dose was evidenced for investigated compound, since the slopes of the lines did not statistically differ (PRE084: −72.42 ± 21.05; RC33: −85.51 ± 15.19; NE100: 19.59 ± 10.13; Compound **1**: −56.37 ± 8.5; compound **2**: −56.98 ± 4.7; compound **3**: −48.00 ± 13.0; *p* = 0.002 NE100 vs. PRE084, RC33, **1**, **2**, **3**, one-way ANOVA). 

**Figure 7:**
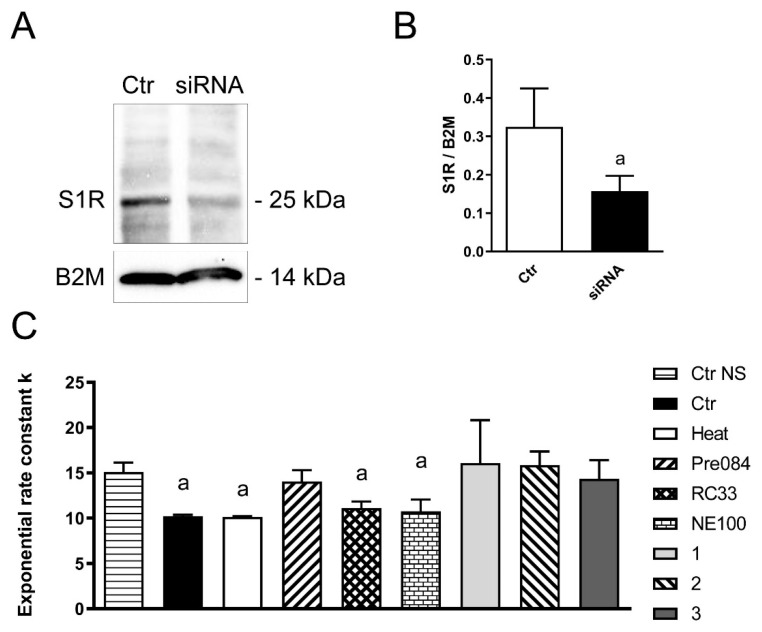
Effect of compounds Pre084, RC33, NE100, **1**–**3** on the water permeability of S1R silenced HeLa cells in heat-stress condition. (**A**) HeLa cells were silenced and the effectiveness in silencing was tested by immunoblotting. A representative blot is shown. 30 μg of proteins were loaded and the blots incubated with anti-S1R antibody. Blots were stripped and re-probed with anti β-2-microglobulin (B2M) antibody. Bands of 25 and 14 kDa were obtained for S1R and B2M, respectively. (**B**) Densitometry was performed by acquiring the blot with the iBright CL1000 imaging system. Semiquantitation was performed by iBA (iBright Analysis Software) and the results were normalized to the corresponding B2M. A significant reduced protein expression in silenced (siRNA) cells compared to controls (scrambled; Ctr) was observed (a, *p* < 0.001; Student’s *t* test). (**C**) Effect of S1R agonist treatment on the water permeability of S1R-KD HeLa cells heat-stressed (Heat). The compounds were used at 20 μM final concentration. Ctr NS, control HeLa cells Not Silenced; Ctr, control, untreated HeLa cells silenced for S1R. Values are means ± SD of 4–15 single shots (time course curves) for each of 4–6 different experiments. a, *p* < 0.05 vs. Ctr NS, Pre084, **1**, **2**, **3** (ANOVA, followed by Newman-Keuls’s *Q* test).

To confirm the results obtained by the co-administration of NE-100 and RC33 and to verify if all the tested S1R agonists act through a S1R-mediated mechanism, experiments on HeLa cells selectively knocked down for S1R have been performed. Short interfering RNAs (siRNAs) targeting human S1R were used. The effectiveness in silencing was assessed by immunoblotting. Figure 7A,B showed that S1R protein was significantly knocked-down of about 50% compared to controls. Successively, silenced HeLa cells were heat-stressed in the presence and in the absence of S1R agonists and the water permeability measured. The water permeability of controls and heat-stressed S1R-KD cells was significantly reduced in comparison with Controls not silenced cells (about 30% reduction), thus demonstrating that S1R depletion induced an oxidative stress even in the absence of heat treatment (Figure 7C). Heat treatment of the S1R-KD cells in the presence of Pre084, **1**, **2** and **3** increased the water permeability from 38 to 58% (Figure 7C). This result demonstrates that the above-indicated compounds were able to counteract the oxidative stress even in S1R-KD cells, likely by a possible interaction with AQPs. On the contrary, pre-treatment of the cells with RC33 did not significantly modify the water permeability, suggesting an exclusive activity of RC33 on S1R. 

**Figure 8:**
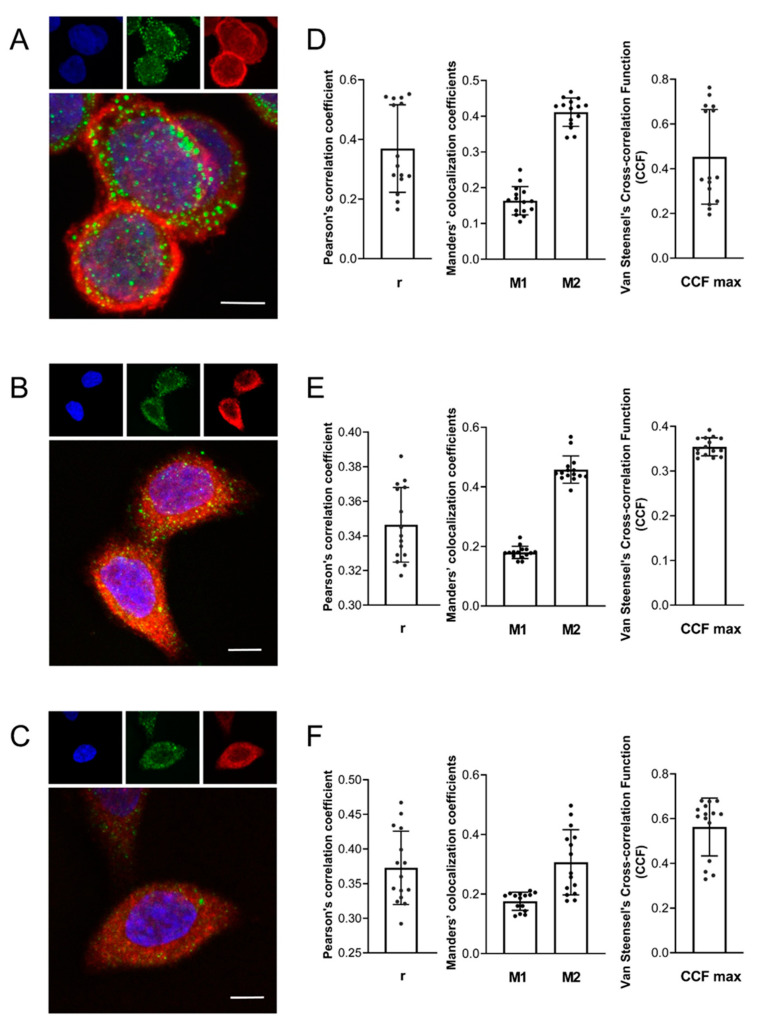
Representative images of confocal laser scanning microscopy and 3D colocalization analysis of AQP3 (**A**,**D**), AQP8 (**B**,**E**) and AQP11 (**C**,**F**) with S1R. Green labeling indicates the presence of S1R, red labeling the expression of AQP3 (**A**), AQP8 (**B**) and AQP11 (**C**), while nuclei were counterstained by Hoechst (blue). Yellow labelling shows colocalization signal of AQP with S1R. Scale bar, 5 μm. (**D**–**F**) Statistical analysis of Pearson’s correlation coefficient r, Manders’ colocalization coefficient (M1 and M2) and Van Steensel’s maxima Cross-Correlation Function (CCF) were obtained from 4 different double immunofluorescence experiments with anti-S1R antibody and anti-AQP3, AQP8 and AQP11 antibodies. Coefficients were determined by 3D analysis for each z-stack (8–15) using the JACoP plugin of Fiji. Each dot of the column represents one single field including different number of cells. The columns represent the mean ± SD of the coefficient values.

Lastly, to evaluate the subcellular localization of AQPs and S1R in HeLa cells as well as their possible interactions, experiments of immunofluorescence staining and confocal microscopy were performed. The results showed in Figure 8A–C evidenced an AQP3 and AQP8 localization mainly in the plasma membrane while AQP11 was confirmed intracellularly [18]. The intracellular labeling of S1R strengthens the hypothesis that the protein resides mainly at the mitochondria-endoplasmic reticulum (ER) interface [24]. No or negligible staining was observed when primary antibodies were substituted with preimmune serum (Figure S1). To evaluate the possible colocalization of AQP3/8/11 with S1R, we analyzed 3D images using JACoP from Fiji to quantify the Pearson’s correlation coefficient r, Manders’ colocalization coefficient (M1 and M2) and Van Steensel’s Cross-correlation Function (CCF). Pearson’s correlation coefficients were included between −0.5 to 0.5, so no conclusions can be drawn [50] (Figure 8D–F).

Manders’ overlap coefficient M1 indicates the percentage of the AQP (red) signal coincident with a S1R signal (green channel) over its total intensity, while M2 indicates the percentage of S1R signal coincident with an AQP signal [51]. The results showed that Manders’ coefficients had a higher overlap of S1R on AQP (M2): 0.458 ± 0.046 (AQP8) > 0.411 ± 0.04 (AQP3) > 0.307 ± 0.109 (AQP11) (Figure 8D–F). The cross-correlation analysis [52] showed bell-shaped curves (Figure S2) with the following CCF maxima values: 0.562 ± 0.129 (AQP11) > 0.453 ± 0.212 (AQP3) > 0.355 ± 0.021 (AQP8) (Figure 8D–F). Taken together, the results suggest at least a partial colocalization of S1R and AQPs, confirming that the herein used cellular model is suitable for studying the effect of S1R modulators on AQP-mediated antioxidant effect.

## 3. Discussion

The main aim of our work was to evaluate the possible involvement of AQP-mediated H_2_O_2_ permeability in the antioxidant effect of S1R modulators. In the first set of experiments, we evaluated the effect of the well characterized S1R agonists PRE084 and racemic RC33 in HeLa cells, and we compared the results with the known S1R antagonist NE100 at a fixed dose. All compounds were tested in the presence and in absence of oxidative stress conditions. Heat shock was used as a cell stressor, and the resulting variation in osmotic water permeability was measured by a stopped-flow light scattering method, which has the advantages of high sensitivity, good reproducibility and cost-effectiveness. We observed that all compounds had no effect on water permeability under normal conditions (non-stressed cells). Conversely, compounds can act on AQPs only in an oxidative environment with two distinct profiles: S1R agonists are able to restore water permeability, whereas the antagonist is ineffective. The subsequent co-administration of RC33 and NE100 to heat-stressed cells, evidenced that the effect of the antagonist quenched the antioxidant properties of the agonist, confirming that the effect appears S1R mediated. Moreover, the effects of racemic RC33 and of (*R*)-RC33 are superimposable, as expected by their S1R binding profile. In fact, our previous studies demonstrated that the racemic RC33 and both (*R*) and (*S*) enantiomers show nearly the same affinity toward the binding site of S1R and are equally effective in promoting neurite outgrowth in PC12 cellular model [49]. Next, we extended our investigation to the S1R ligands **1**–**3**. It is worth noting that compounds **1**–**3** were originally designed by merging the pharmacophoric elements of different neuroprotective and/or antioxidant molecules. Again, heat-stressed cells treated with compounds **1**–**3** (20 μM) displayed a restored water permeability.

A dose-response correlation has been determined for all the compounds, assessing the cellular H_2_O_2_ content. All compounds with S1R agonist profile show antioxidant activity mainly related to the recovery of AQP permeability. However, at micromolar concentrations, the S1R agonist might interact with off-targets. Once the best compound will be selected, a complete binding profile will be drawn.

To understand if the effect is only due to S1R interaction, we performed experiments in HeLa cells knocked down for S1R. The results clearly showed that RC33 is unable to recover S1R-depleted cells from oxidative stress and thus it acts only through S1R modulation. Conversely, Pre084, **1**, **2** and **3** are still able to increase ROS scavenging thereby protecting the cells from oxidative stress damage. Lastly, to evaluate the possibility of interactions between AQPs and S1R we have performed double immunofluorescence experiments. As highlighted in the introduction section, S1R resides mainly at the interface mitochondrion associated membrane of the ER [24]. Moreover, AQP8 and AQP11 are localized in mitochondria [25,26] and ER [18] respectively, but ER can change is shape forming a reticular structure throughout the entire cytoplasm, thus enabling physical connections to other subcellular structures, such as the plasma membrane and mitochondria [53,54]. Results of co-localization experiments evidenced an intracellular localization of S1R [24] and AQP11 [32], while AQP3 and AQP8 were localized mainly on the plasma membrane. The colocalization analysis evidenced a partial colocalization and/or contiguity of S1R with the investigated AQPs. Therefore, the herein used cellular model can represent a useful model for studying the effects of S1R modulators on AQP-mediated water and H_2_O_2_ permeability and for the development of novel therapeutic approaches targeting oxidative stress. Moreover, an interaction of S1R with the inositol triphosphate receptor, with the potassium channel Kv1.4 and with other proteins has been previously observed, supporting the possibility of an interaction between S1R and other contiguous client proteins such as AQPs [27].

## 4. Materials and Methods

### 4.1. Chemicals and Reagents

Reagents and solvents for synthesis, TLC and NMR were purchased from Sigma Aldrich. Silica gel for flash chromatography (60 Å, 230–400 Mesh) was purchased from Sigma Aldrich. Solvents were evaporated at reduced pressure with the Heidolph Laborota 4000 Efficient equipment. Analytical thin layer chromatography (TLC) analyses were carried out on silica gel pre-coated glass-backed plates (TLC Silica Gel 60 F254, Merk) impregnated with a fluorescent indicator, and visualised with the instrument MinUVIS, DESAGA^®^ Sastedt-GRUPPE by ultraviolet (UV) radiation from UV lamp (λ = 254 and 366 nm) or by stain reagents such as Ninidrine and Cerium Molybdate. NMR were measured at room temperature (15 °C–25 °C) on a Bruker Advance 400 MHz spectrometer, using tetramethylsilane (TMS) as internal standard and a BBI 5 mm probe. All raw FID files were processed with Top Spin program from Bruker and the spectra analysed using the MestRenova 6.0.2 program from Mestrelab Research S.L. Chemical shifts are expressed in parts per million (ppm, δ scale). 1H-NMR spectroscopic data are reported as follow: chemical shift in ppm (multiplicity, coupling constants J (Hz), integration intensity). The multiplicities are abbreviated with s (singlet), d (doublet), t (triplet), q (quartet), m (multiplet) and brs (broad signal). The chemical shift of all symmetric signals is reported as the centre of the resonance range. ^13^C-NMR spectroscopic data are reported as follows: chemical shift in ppm. UPLC-UV-ESI/MS analyses were carried out on a Acuity UPLC Waters LCQ FLEET system using an ESI source operating in positive ion mode, controlled by ACQUIDITY PDA and 4 MICRO (Waters). Analyses were run on a ACQUITY BEH Phenyl (ABP) (50 × 2.1 mm, 1.7 µm) or ACQUITY BEH Shield (ABS) (100 × 2.1 mm, 1.7 µm) columns, at room temperature, with gradient elution (solvent A: water containing 0.1% of formic acid; solvent B: methanol containing 0.1% of formic acid; gradient: 10% B in A to 100% B in 3 min, followed by isocratic elution 100% B for 1.5 min, return to the initial conditions in 0.2 min) at a flow rate of 0.5 mL min^−1^. All the final compounds had 95% or greater purity.

#### Compound Synthesis

**Scheme 1:**
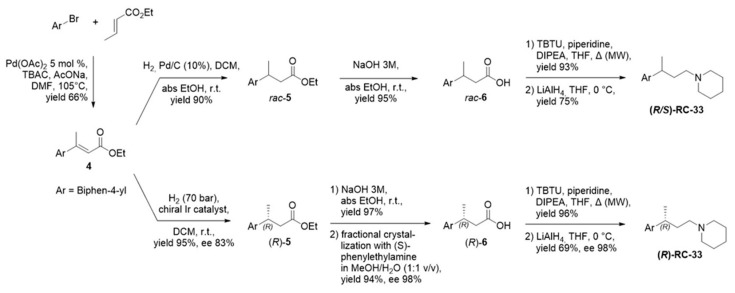
Schematic synthesis procedures of racemic and enantiomeric RC33.

Racemic and enantiomeric RC33 have been re-synthetized according to the already published procedures [42,49]. These are summarized in Scheme 1. 

Briefly, a Heck reaction between 4-Bromobiphenyl and ethyl crotonate was performed to access α, β-unsaturated ester **4**. This was subjected to a reduction of the double bond, performed either without stereocontrol (H_2_ gas in presence of Pd/C) or under enantioselective conditions (H_2_ gas in presence of a chiral Ir catalyst), to access racemic **5** and (*R*)-**5** respectively. Ester hydrolysis afforded acid **6**, which could be subjected to fractional crystallization to enhance the enantiomeric excess in the synthesis of enantiopure RC33. Acid **6** was then subjected to amidation with piperidine in the presence of condensing agent TBTU. Afterwards, reduction of the amide moiety with LiAlH_4_ afforded RC33 either in racemic or enantiopure form (ee 98%). The identity of obtained products was confirmed by ^1^H- and ^13^C-NMR, whereas optical purity was assessed by chiral-HPLC following the method developed in our lab. Overall, the analyses resulted consistent with published data [41,55]. 

**Scheme 2:**
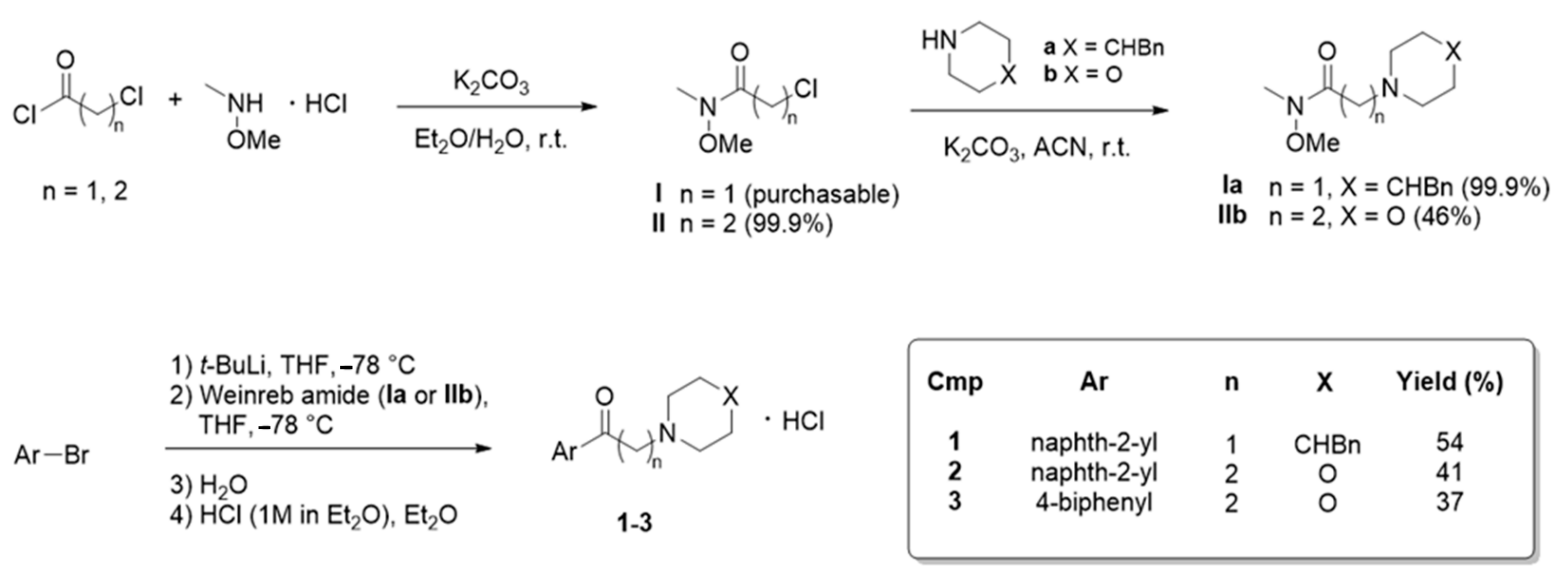
Schematic synthesis procedures of compounds **1**–**3**.

Compounds **1**–**3** were prepared following the procedure reported in our previous publication, with slight modifications [45]. Overall, the synthetic strategy is based on the Weinreb ketone synthesis, as shown in Scheme 2.

Briefly, Weinreb amides **I**,**II** were either purchased if commercially available (amide **I**) or prepared by reacting the corresponding acyl chloride with *N*,*O*-dimethylhydroxyamine hydrochloride. Afterwards, nucleophilic substitution on Weinreb amides **I**,**II** was carried out using amines **a**,**b**. This led to the key intermediates **Ia**–**IIb**. Finally, compounds **1**–**3** were obtained by coupling intermediates **Ia**–**IIb** with different aryl lithium species generated in situ. Aromatic moieties thus introduced are naphth-2-yl and 4-biphenyl. This last step involves the smooth bromo-lithium exchange on the aryl bromide to access the lithiated arene that, after addition of Weinreb amides **Ia**–**IIb** and quenching with H_2_O gave the desired crude ketones **1**–**3** in good yields. After flash chromatography purification, compounds were converted into their corresponding hydrochlorides by addition of HCl in Et_2_O. The identity of obtained products was confirmed by ^1^H- and ^13^C-NMR and the analyses resulted consistent with published data [45].

### 4.2. Cell Culture

HeLa cells were grown in plastic tissue culture flasks using Dulbecco’s modified minimal essential medium high glucose, supplemented with 10% fetal bovine serum, 1% L-glutamine, 1% penicillin and streptomycin and maintained at 37 °C in a humidified atmosphere of 5% CO_2_, 95% air.

### 4.3. Water Permeability Measurements

Osmotic water permeability was measured in HeLa cells suspension by the stopped-flow light scattering method as previously described [20]. The experiments were performed at 25 °C on a stopped flow apparatus (RX2000, Applied Photophysics, Leatherhead, UK) with a pneumatic drive accessory (DA.1, Applied Photophysics) straightforward coupled with a Varian Cary 50 spectrometer (Varian Australia Pty Ltd., Mulgrave, Australia). Scattered light intensity with a dead time of 6 ms was recorded at a wavelength of 450 nm. The time course of cell swelling caused by exposure to the hypotonic gradient (150 mosm/L) was measured for 60 s at the acquisition rate of one point/0.0125 s. The initial rate constant of cell volume changes (k) was obtained by setting the time course light scattering with a single exponential equation (GraphPad Prism 4.00, 2003). Representative single scattering traces in the different experimental conditions and the results of the fitting were shown in Figure S3.

To evaluate the antioxidant effect of the test compounds on water permeability HeLa cells were divided into different groups: (1) controls, cells left at room temperature (21 °C) in the presence of the same concentration of methanol than treated cells; (2) heat-stressed cells, cells subjected to heat-treatment by placing them in a water thermostatic and shacking bath at 42 °C for 3 h; (3) heat-stressed cells pre-treated, cells heat-stressed with the antioxidants compounds at 20 µM final concentration (dissolved in methanol). Moreover, to test the possible capacity of the molecules to affect the AQP gating in eustress condition, HeLa cells were treated in the presence and in the absence of the compounds by incubating at 21 °C for 3 h. In preliminary experiments, osmotic water permeability was measured at both 37 °C and 21 °C to check that the cells responded in optimal conditions. No differences in osmotic permeability were observed (not shown).

### 4.4. Hydrogen Peroxide Permeability Measurements

Dose-response relationship was assessed for all the compounds tested by measuring the hydrogen peroxide concentrations in heat-stressed HeLa cells. Hydrogen peroxide levels were measured by a fluorescence method using the 5-(and-6)-chloromethyl-20,70-dichlorodihydro-fluorescein diacetate, acetyl ester reagent (CM-H2DCFDA) (Thermo Fisher Scientific Inc., Monza MB, Italy) as previously described [20]. Briefly, cells were centrifuged at 200 rcf for 5 min. The cell pellet was resuspended in PBS with increasing concentrations of the compounds (0, 5, 10, 20, 40 μM final concentration) and subjected to heat-stress as above indicated. Before terminating the incubation, the CM-H2DCFDA reagent was added at 10 μM final concentration and left for further 15 min at 42 °C. Then, cells were centrifuged, and the pellet resuspended in PBS. Hydrogen peroxide levels were measured by using a CLARIOstar^®^ microplate reader (BMG LABTECH, Ortenberg, Germany). Values are expressed as arbitrary unit per mg total protein.

### 4.5. Gene Silencing 

S1R knockdown was performed by treating HeLa cells with ON-TARGETplus SMARTpool Human SIGMAR1 siRNA (FE5LHUMANXX0005; Carlo Erba Reagents Srl, Cornaredo, Milan, Italy) at a 25 nM final concentration. Negative controls were done with scrambled siRNA. siRNAs were diluted in siRNA Diluition Buffer (N0413, Sigma-Aldrich, Milan, Italy) and mixed with N-TER peptide (N2788, Sigma-Aldrich, Milan, Italy) pre-diluted in PBS, according to the manufacturer’s instructions to create the Target siRNA Nanoparticle Formation Solution (NFS). When HeLa cells reached 50% confluence, medium was removed and replaced with fresh medium containing NFS. After 30 min incubation at 37 °C, the NFS was diluted in the culture medium and added to the cells and incubated at 37 °C for 24 h. 

Immunoblotting was used to validate the gene silencing and the silenced cells were used 24 h after transfection. S1R protein quantification was performed in four independent knockdown experiments.

### 4.6. Protein Content

The protein content was determined with the Bradford method [56], using bovine serum albumin as standard.

### 4.7. Immunoblotting

Cells were homogenized with a Dounce homogenizer in RIPA buffer (150 mM NaCl, 0.5% sodium deoxycholate, 0.1% SDS, 0.1% Triton X-100, 50 mM Tris-HCl, pH 8) supplemented with a protease inhibitor cocktail (cOmplete Tablets EASYpack, 04693116001, Roche, Monza MB, Italy). 

Immunoblotting was carried out as previously described [57] loading 30 µg proteins. Membranes were incubated overnight with anti-Sigma1 Receptor (B-5) (sc-137075, 1:500 dilution; Santa Cruz Biotechnology, Inc., Heidelberg, Germany) in a blocking solution (Tris buffered saline, 5% skimmed dry milk and 0.1% Tween). The membranes were washed thrice and incubated for 1 h with peroxidase-conjugated rabbit anti-mouse IgG (Dakocytomation, P0260, Agilent, Cernusco sul Naviglio MI, Italy), diluted 1:120,000 in blocking solution. The bands were detected with Westar Supernova western blotting detection system (CYANAGEN) and pre-stained molecular weight markers (ab116028, Abcam, Cambridge, UK) used to calculate the molecular weights of the bands.

Blots were stripped following Yeung and Stanley [58] and reprobed with anti β-2-microglobulin (B2M) rabbit antibody (ab75853, Abcam, Cambridge, UK) diluted 1:10,000 in blocking solution.

Densitometry was performed by acquiring the blots with the iBrightTM CL1000 Imaging System (Thermo Fisher Scientific Inc., Monza MB, Italy). The semiquantitation of the bands was performed using the iBA (iBright Analysis Software; Thermo Fisher Scientific Inc., Monza MB, Italy) and the results were expressed as S1R/B2M ratio.

### 4.8. Immunofluorescence Staining, Confocal Microscopy and Colocalization Analysis

Immunolocalization of AQP3, 8, 11 and S1R was evaluated in HeLa cells seeded on glass coverslips overnight, fixed with 4% paraformaldehyde in PBS for 30 min, and then washed with PBS. Antigen retrieval was performed by placing the glass cover slips in petri dishes containing retrieval buffer (0.05% tween-20, 10 mM citrate-HCl buffer, pH 6.0) in an oven at 80 °C for 30 min. The coverslips were washed with PBS and then blocked with 3% BSA in PBS at room temperature for 30 min. Double labeling experiments were performed by incubating the coverslips overnight (in the cold) with affinity pure anti-AQP3 or AQP8 or AQP11 primary antibodies (Anti-AQP3, ab125045, Abcam, Cambridge, UK; Anti-AQP8, HPA046259, Sigma-Aldrich, Milan, Italy; Anti-AQP11, ab122821, Abcam, Cambridge, UK) and with anti-Sigma1 Receptor (B-5) (sc-137075, 1:250 dilution; Santa Cruz Biotechnology, Inc., Heidelberg, Germany). Anti-AQP antibodies were used at the following dilutions in antibody diluent (Dako): AQP3, 1: 400; AQP8 1: 500; AQP11, 1:100. After three 5 min washes with PBS, coverslips were incubated at room temperature with the fluorescent secondary antibody (ab150117, 1: 400, Abcam, Cambridge, UK) and rhodamine red coniugated Fab donkey anti-rabbit IgG (H + L) (1:1000 dilution; 811-7002; Rockland Immunochemicals, distributred by tebu-bio s.r.l.,Magenta MI, Italy) for 30 min.

Slides were then washed 3 × 5 min with PBS, nuclei were staining with Hoechst 33342 and after washing trice with PBS mounted with BrightMount/Plus Aqueous Mounting Medium (ab103748, Abcam, Cambridge, UK). Slides were examined with a TCS SP5 II confocal microscopy system (Leica Microsystems, Buccinasco MI, Italy) equipped with a DM IRBE inverted microscope (Leica Microsystems, Buccinasco MI, Italy) and images visualized and analyzed by LAS AF software (Leica Microsystems Application Suite Advanced Fluorescence, Buccinasco MI, Italy). Control experiments were performed simultaneously using non-immune serum.

To evaluate the colocalization, we used JACoP (just another Colocalization Plugin) from Fiji to quantify the Pearson’s correlation coefficient r, Manders’ colocalization coefficient (M1 and M2) and Van Steensel’s Cross-correlation Function (CCF) of 3D images [50,51,52].

### 4.9. Statistics

All data were expressed as mean ± SD. The significance of the differences of the means was evaluated by using repeated measures one-way ANOVA followed by Newman-Keuls’s *Q* test. All statistical tests were carried out using GraphPad Prism 4.00, 2003.

## 5. Conclusions

The antioxidant properties of the investigated S1R agonists rely on a double mechanism: the interaction with S1R and the modulation of AQP-mediated H_2_O_2_ permeability, with the only exception of the selective S1R agonist RC33. Although S1R are undoubtedly involved in the oxidative stress process, the roles of S1R are not completely understood and thus results herein presented add a piece to the complex puzzle of S1R. S1R has been extensively studied in the past decades, and its modulators have been proposed as viable tools for different therapeutic applications, reaching advanced stages of drug development (e.g., MR309, a Sigma-1 antagonist is currently in Phase II clinical trial for the treatment of neuropathic pain) [33,59,60]. On the other hand, aquaporins are still largely unexplored from a medicinal chemistry standpoint and have been recognized as druggable molecular targets only recently [20,61,62,63,64,65,66]. 

In this paper, we reported for the first time a series of compounds that are able to exert effects on both S1R and AQP-mediated H_2_O_2_ permeability, suggesting that there is a mutual correlation between the two targets. Modulation of both S1R and AQP permeability may be exploited as a synergic strategy to achieve antioxidant effects and enhanced therapeutic potential. Hence, the class of aryl-aminoalkyl-ketones herein investigated holds promise for small-molecule-based therapy to treat diseases involving oxidative stress, namely neurodegenerative diseases and cancer. 

## Supplementary Materials

The following are available online at https://www.mdpi.com/article/10.3390/ijms22189790/s1. Figure S1: Representative traces of stopped-flow osmotic water permeability measurements obtained in the different experimental conditions; Figure S2: Immunofluorescence negative control; Figure S3: Representative Van Steensel’s plots for AQP3, AQP8, AQP11, and S1R.
