# Supplementary material for: Sigma-1 Receptor Agonists Acting on Aquaporin-Mediated H2O2 Permeability: New Tools for Counteracting Oxidative Stress
Source: Int J Mol Sci. 2021 Sep 10;22(18):9790. doi: 10.3390/ijms22189790 (PMC8467392; doi:10.3390/ijms22189790)
Supplement: Supplementary file 1 [file ijms-22-09790-s001.zip › ijms-1280658-supplementary.pdf]

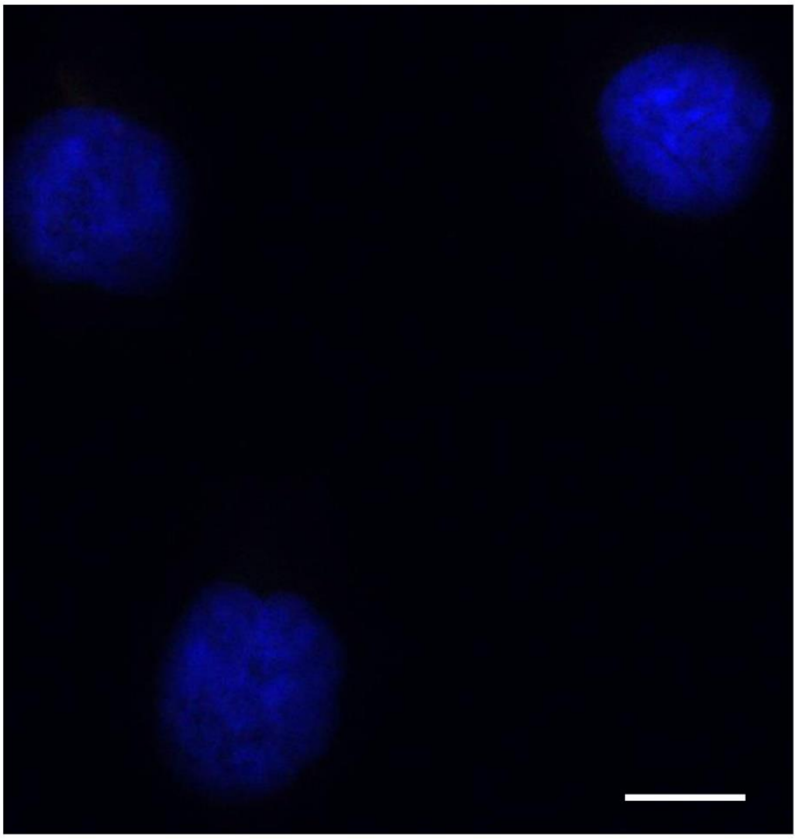

Figure S1. Immunofluorescence negative control. No or faint staining was observed when anti-aquaporins and anti-S1R antibodies were substituted with preimmune serum. Nuclei were counterstained by Hoechst (blue). Scale bar, 5 μm.

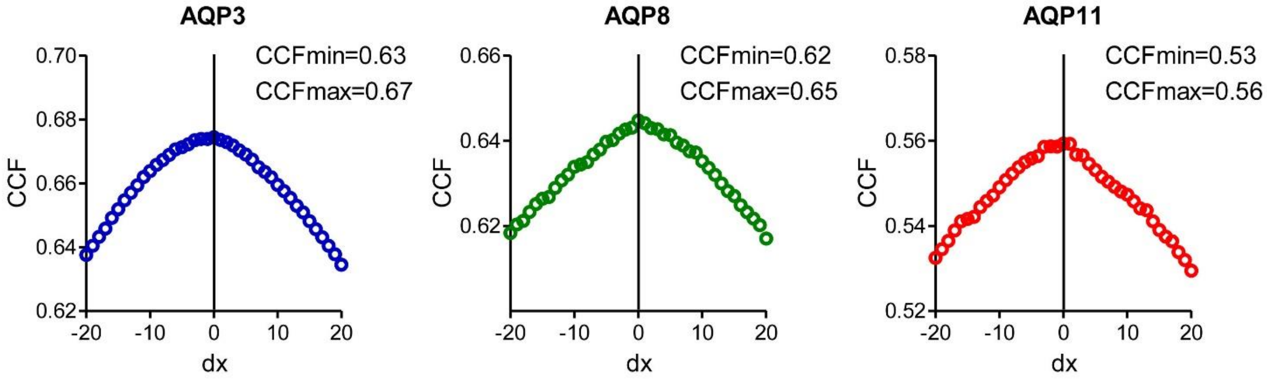

Figure S2. Representative Van Steensel's plots for AQP3, AQP8, AQP11 and S1R. Van Steensel's plots give a cross-correlation coefficient (CCF) plotted against lateral delocalization (dx = 0 to ±20 pixel). Minima and maxima of cross-correlation coefficient plots are given in the legend for each plot.

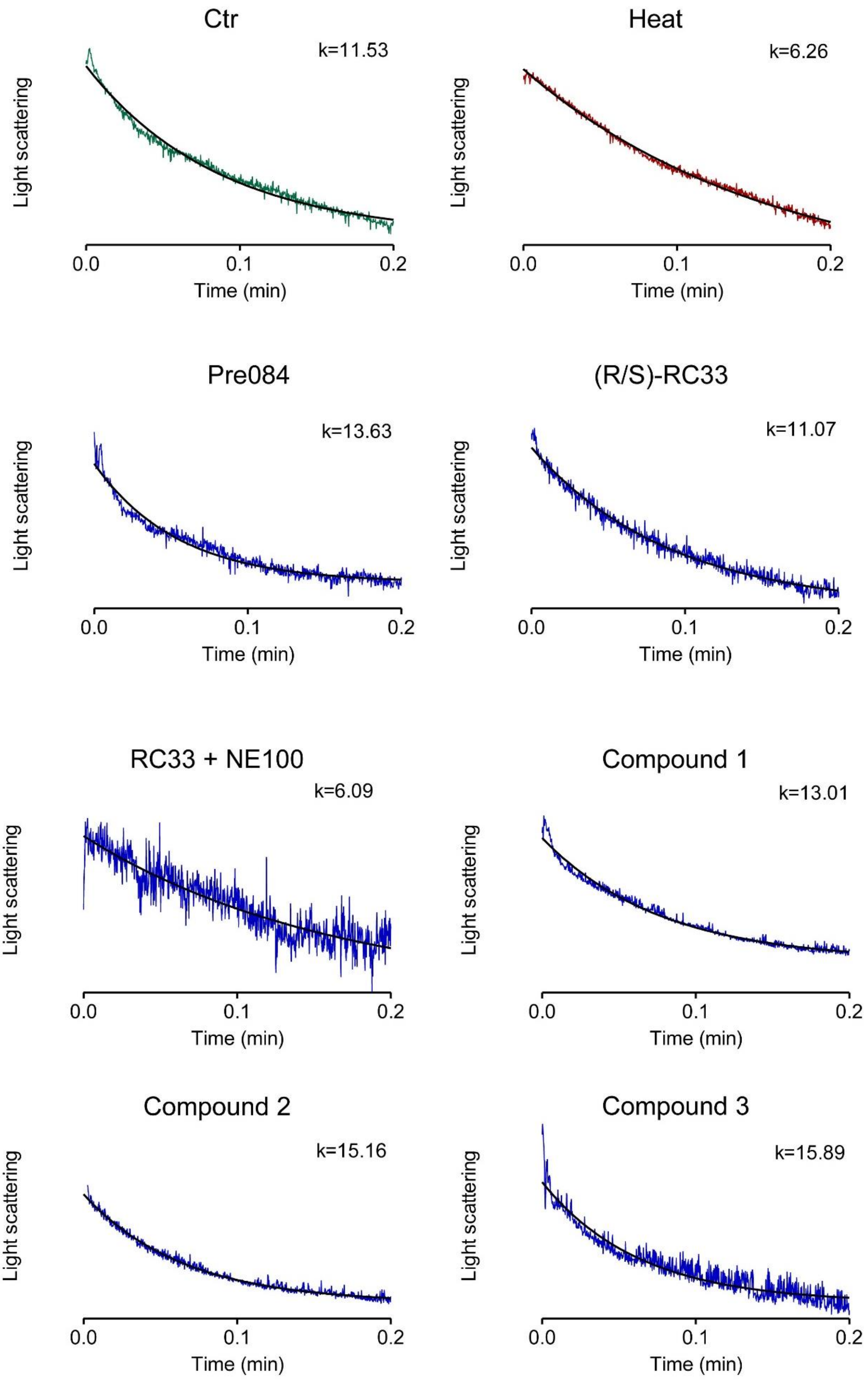

Figure S3. Representative traces of stopped-flow osmotic water permeability measurements obtained in the different experimental conditions. HeLa cells were exposed to a 150 mOsm osmotic gradient in the following conditions: untreated cells (Controls; Ctr), cells heat-stressed (Heat), heat-stressed cells pre-treated with test compounds Pre084, (*R*/*S*)-RC-33, NE-100, co-administration of (R/S)-RC-33+NE100 ((RC33+NE100), Compounds 1, 2, 3. Exponential rate constant k values of single curves and the results of the fitting are also shown.
